# Use of an Innovative Personality-Mindset Profiling Tool to Guide Culture-Change Strategies among Different Healthcare Worker Groups

**DOI:** 10.1371/journal.pone.0140509

**Published:** 2015-10-21

**Authors:** M. Lindsay Grayson, Nenad Macesic, G. Khai Huang, Katherine Bond, Jason Fletcher, Gwendolyn L. Gilbert, David L. Gordon, Jane F. Hellsten, Jonathan Iredell, Caitlin Keighley, Rhonda L. Stuart, Charles S. Xuereb, Marilyn Cruickshank

**Affiliations:** 1 Infectious Diseases and Microbiology Department, Austin Health, Heidelberg, Melbourne, Australia; 2 Hand Hygiene Australia, Australian Commission on Safety and Quality in Health Care, Melbourne, Australia; 3 Department of Epidemiology & Preventive Medicine, Monash University, Melbourne, Australia; 4 Department of Medicine, University of Melbourne, Melbourne, Australia; 5 Infection Prevention and Control Department, Bendigo Health, Bendigo, Australia; 6 Centre for Infectious Diseases & Microbiology, ICPMR Westmead Hospital, Sydney, Australia; 7 Marie Bashir Institute for Infectious Diseases and Biosecurity, University of Sydney, Sydney, Australia; 8 Department of Microbiology and Infectious Diseases, SA Pathology, Flinders Medical Centre, Adelaide, Australia; 9 Infection Control & Infectious Diseases Departments, Monash Health, Monash University, Clayton, Australia; 10 XAX Pty. Ltd., Melbourne, Australia; 11 Australian Commission on Safety and Quality in Health Care, Sydney, Australia; University of Calgary, CANADA

## Abstract

**Introduction:**

Important culture-change initiatives (e.g. improving hand hygiene compliance) are frequently associated with variable uptake among different healthcare worker (HCW) categories. Inherent personality differences between these groups may explain change uptake and help improve future intervention design.

**Materials and Methods:**

We used an innovative personality-profiling tool (ColourGrid^®^) to assess personality differences among standard HCW categories at five large Australian hospitals using two data sources (HCW participant surveys [PS] and generic institution-wide human resource [HR] data) to: a) compare the relative accuracy of these two sources; b) identify differences between HCW groups and c) use the observed profiles to guide design strategies to improve uptake of three clinically-important initiatives (improved hand hygiene, antimicrobial stewardship and isolation procedure adherence).

**Results:**

Results from 34,243 HCWs (HR data) and 1045 survey participants (PS data) suggest that HCWs were different from the general population, displaying more individualism, lower power distance, less uncertainty avoidance and greater cynicism about advertising messages. HR and PS data were highly concordant in identifying differences between the three key HCW categories (doctors, nursing/allied-health, support services) and predicting appropriate implementation strategies. Among doctors, the data suggest that key messaging should differ between full-time *vs* part-time (visiting) senior medical officers (SMO, VMO) and junior hospital medical officers (HMO), with SMO messaging focused on evidence-based compliance, VMO initiatives emphasising structured mandatory controls and prestige loss for non-adherence, and for HMOs focusing on leadership opportunity and future career risk for non-adherence.

**Discussion:**

Compared to current standardised approaches, targeted interventions based on personality differences between HCW categories should result in improved infection control-related culture-change uptake. Personality profiling based on HR data may represent a useful means of developing a national culture-change “blueprint” for HCW education.

## Introduction

“Would you use the same marketing strategy to sell a Volvo to a nurse as you would if you were selling it to a doctor? Of course not! So why are you surprised that hand hygiene compliance rates are worse among doctors than nurses?” (Exasperated comment from an advertising executive consulted by Hand Hygiene Australia).

Infection prevention interventions have repeatedly been shown to decrease mortality, yet uptake by healthcare workers (HCWs) has often been suboptimal and doctors are known to be sceptical about guidelines generally [1–11]. Despite socioeconomic, cultural and educational differences between various HCW groups, such factors are rarely taken into account when designing multimodal culture-change strategies; however, these differences may have an important impact on behavior change and the success of such interventions [6,7,12].

Although social marketing, based on market research and segmentation, is increasingly used to influence a variety of health behaviors in the general community (e.g. smoking, seatbelt use and physical exercise), there has been limited use of this approach in healthcare [13]. Perhaps one reason is the resource-intensive nature of qualitative approaches, which frequently require the use of focus groups, structured interviews and detailed surveys [14].

We used an innovative personality profiling tool (ColourGrid^®^, see Methods) to assess differences between HCWs using data derived directly from HCW surveys, as well as basic information derived from large institutional human resources (HR) databases at participating sites. Our aims were to:

1. Identify any generic personality differences between HCWs and the general Australian population to guide overall intervention strategies for HCWs
2. Identify any differences between HCW groups
3. Compare the accuracy of profiling using non-identifying HR data to that obtained directly from individual HCWs who completed a formal ColourGrid survey.
4. Develop a generic “blueprint” of optimal educational and marketing approaches to improve uptake of culture-change initiatives among HCWs, using three specific examples (hand hygiene compliance; antibiotic stewardship and adherence to isolation protocols for patients with multidrug-resistant organisms [MROs]).

## Materials and Methods

### Description of Personality Profiling tool—ColourGrid^®^

**Fig 1:**
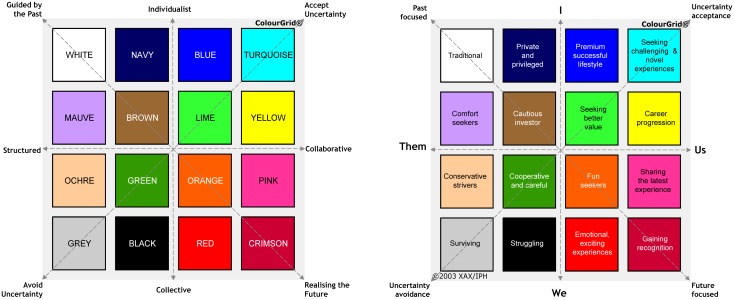
ColourGrid^®^ principles and summary profiles. The principles of ColourGrid are based on Hofstede’s cultural dimensions theory (see text). The principles include: **Power distance:** relates to the extent to which the less powerful members of organizations and institutions accept and expect that power is distributed unequally. It suggests that a society’s level of inequality is endorsed by the followers as much as by the leaders. **Uncertainty avoidance:** indicates to what extent society tolerates uncertainty and ambiguity, and it shows how comfortable its members feel in unstructured situations which are novel, unknown, surprising or different from usual. **Individualism:** is the degree to which individuals are integrated into tight groups (collectivist) or loose groups (individualist). **Long-term orientation:** reflects long-term pragmatic attitudes versus short-term normative attitudes. Cultures scoring high on this dimension show emphasis on future rewards, notably saving, persistence, and adapting to changing circumstances.

ColourGrid is a personality and marketing research tool [15] which aims to identify the social, cultural, economic and behavioural factors (often referred to as “mindset” [15]) that influence peoples’ retail and business choices and thus allows market segmentation. The tool’s framework is based on Hofstede’s highly cited Cultural Dimensions Theory [16,17], with values being plotted on four different cultural dimensions (individualism *vs* collectivism, power distance index, uncertainty avoidance index, long-term orientation *vs* short-term orientation) (see Fig 1) which have been used to explain some inter-country differences in healthcare, but have not been used to guide social marketing and culture-change among HCWs [18,19].

The ColourGrid system links the Hofstede approach with data obtained from two sources: the Australian National Census (population– 18,339,443; 7,144,096 dwellings/households) [20], which defines household characteristics, and from Roy Morgan Research, an Australian company that has regularly conducted detailed direct consumer marketing research over the past 70 years on social, political and economic trends [21]. These data have been used to create a computerized algorithm that generates personality scores across 16 different domains, against which market-derived colour preferences have been assigned (see Fig 1 and S1 Table for typical colour profiles). Given the usual commercial intent of ColourGrid, profiling has typically focused on residential geographical locality (postcode, suburb) within Australia, but has also been used to improve uptake of various community-wide healthy lifestyle initiatives [22]. However, completion of a formal ColourGrid survey allows very accurate profiling without knowledge of residential address. These features of ColourGrid provide the option of assessing the comparability of profiles generated entirely from basic non-identifying demographic data obtained from a hospital’s HR department with those obtained from direct participant surveys (PS) to help define improved marketing strategies for key healthcare culture-change initiatives. Should HR-derived profiles prove similar to those obtained from PS data, this could support the concept of developing a national profile of various HCW groups and an action “blueprint” for intervention design that is based solely on easily obtainable HR data.

For each of the 16 ColourGrid domains, results are displayed as a colour matrix (Fig 1) with scores compared to the mean value for that feature in the comparator population (mean value assigned as 100%). Thus a domain score that is higher than the population mean score for that domain is depicted as a coloured box, whereas a score that is lower than the mean is depicted as a circle, with the size of the box/circle linearly proportional to the percentage difference from the mean (see S1 Fig; lower and upper limits of depiction 50% and 150%, respectively). Where scores were outside these limits, the exact percentage is cited below the relevant box/circle. This approach allows for the ready visual display of multiple personality features simultaneously and assists in appreciating the overall personality profile in a novel multi-dimensional manner.

By definition, results vary according to the comparator group used, but this approach also allows for “normalisation” of results within groups. For instance, one can compare the differences between various HCW categories by normalising the mean results for the total HCW cohort to 100%, then analyse the results for individual categories against the normalised values. This potentially allows subtle differences to be identified.

### Study sites

The study was undertaken at 5 major public hospitals (four urban, one regional) in three Australian States—each had active infectious diseases/infection control teams with an interest in innovative culture-change initiatives. The study was approved by the relevant Human Research Ethics Committee at each site (Human Research Ethics Committee (HREC)—Low & Negligible Risk Research (LNRR), Department of Health, State Government Victoria. Site specific assessments (SSA)–Austin Health, Monash Health, Bendigo Health; South Adelaide Clinical Human Research Ethics Committee, South Adelaide Health Service, Government of South Australia. SSA—Flinders Medical Centre.; Western Sydney Local Health District HREC, LNRR, NSW Government. SSA—Westmead Hospital) using the same informed consent conditions for all survey participants. Implied consent was provided when participants logged onto the specific study website, provided a personal email address to which they were sent a link to the ColourGrid questionnaire. Participants used the link to complete the questionnaire and then received their personality profile assessment electronically via a return email to their nominated email address.

### Healthcare worker categories

All sites used the same national eight-tiered HCW category classification: nursing, administrative and clerical, medical support, hotel and allied, full-time senior medical officer (SMOs), part-time senior medical officer (‘visiting medical officers’ or VMOs), hospital medical officers (HMOs) and ancillary support (see Table 1 for detailed definitions) [23]. If a site was found to have varied from this classification structure, data from the relevant HCW group was allocated into the appropriate national category for analysis. For practical messaging purposes we also analysed results according to three combined occupational categories, according to the level of direct clinical patient contact: doctors (SMOs, VMOs, HMOs), nursing-allied health (nursing and ancillary support) and support services (administration and clerical, hotel and allied services and medical support services) (see Table 1). Thus, results were analysed according to all eight HCW categories and the three combined clinical-contact (CC) categories.

**Table 1:** Description of the eight national HCW categories and the combined three clinical-contact (CC) categories.

HCW Category	Detailed description and features	Clinical-contact Category
Senior Medical Staff—Full-time (SMO)	• Senior clinicians full-time employed by the hospital	Doctors
	• Many with honorary university appointments	
	• Highly sought after academic position	
Senior Medical Staff—Part-time (VMO)	• Senior clinicians who generally have substantive private practices, but who are employed part-time (usually 0.1–0.3) by the hospital to provide inpatient and outpatient care	
	• Some have honorary academic university appointments	
	• Higher hourly pay rate than SMOs but hospital position generally less secure	
Hospital Medical Officers (HMO)	• Interns, residents, Registrars/Fellows	
	• Undergoing post-graduate training	
	• Vast majority employed full-time by hospital on an annual contract basis	
Nursing Services	• All nurses, regardless of specialist training or seniority	Nursing-Allied Health
	• Includes small number of nurse practitioners	
	• Mixture of full-time and part-time appointments	
Ancillary Support	• Allied health staff, including physiotherapists, occupational therapists, dieticians	
	• Mixture of full-time and part-time appointments	
Medical Support Services	• Technical staff including laboratory technicians, pharmacists, radiographers	Support Services
	• Mixture of full-time and part-time appointments	
Admin and Clerical	• Administrative staff—clerks, secretaries, personal assistants	
	• Mixture of full-time and part-time appointments	
Hotel and Allied Services	• Staff involved in logistical and maintenance activities—cleaning, food preparation and delivery, security	
	• Mixture of full-time and part-time appointments	

### Data sources

The HR departments at each study site provided the following de-identified information regarding all employees: age, gender, suburb and postcode of home residence, employment status (full-time, part-time) and HCW category. Employee data was excluded from further analysis if the HR residential address information was invalid (e.g. invalid postcode, non-Australian home address), or if no HCW category was specified.

HCW employees were recruited from each of the study sites to formally complete a ColourGrid survey. This was undertaken within four weeks of HR data download at each site, to ensure that survey participants were included in the HR data. A standardised 8–10 week campaign to recruit participants was undertaken at each site. A secure site-specific survey link was established on the Hand Hygiene Australia website to allow participants to complete a ColourGrid survey on-line [24] (see S2 Fig for questionnaire details), with the individual’s profile results subsequently emailed to the participant’s specified personal email address—thereby retaining individual confidentiality. In registering for the survey, participants specified their HCW category and provided consent, but were not required to disclose detailed identifying information such as residential suburb or postcode.

### Data analysis

To ensure blinding, all HR- and PS-derived data was coded (by MLG, KB, GKH) so that the HCW category was unknown to the team member (CSX) who analysed results and derived the predicted profiles. Post-analysis, these codes were broken to allow assignment of personality profiles to the relevant HCW (and CC) category. Both HR- and PS-derived profiles were compared for consistency across study sites. Statistical analysis was by either Chi-square or t-test, as appropriate.

### HR-derived data

Using the ColourGrid national database as the comparator, HR-derived data was assessed for each site and collectively to provide predicted personality profiles for all HCWs collectively compared to the overall Australian population and for each HCW (and CC) category. HR data were also “normalised” (see above) to allow a comparison of predicted profiles with those derived from PS-derived data, since PS data did not include residential information (suburb, postcode) and could therefore not be validly assessed using national comparator information.

### Participant Survey data

PS data were normalised then derived personality profiles, both overall and for each HCW (and CC) category, were compared to those predicted from the HR-derived data. Detailed analysis of PS data for each of the three doctor categories (SMO, VMO, HMO) was undertaken to assess whether any subtle differences could be identified that might inform intervention strategies.

### Application of personality profiling to specific infection control strategies

To help translate the value of our study findings into potentially meaningful action, we used derived personality profiles for each CC categories and the three medical categories to guide proposed marketing strategies (by CSX) to improve uptake in three specific infection control initiatives. These initiatives were: improving hand hygiene compliance, improved antibiotic prescribing/stewardship and improved adherence to strict isolation procedures for patients infected/colonised with MROs. These examples were selected because they were each known to be difficult to implement [14,24,25] and associated with different levels of impact on doctors, patients and the community (see S2 Table).

### Funding

This work was supported by Hand Hygiene Australia and the Australian Commission on Safety and Quality in Health Care via a contract with Austin Health. The marketing research company XAX Pty. Ltd., (Melbourne, Australia) was contracted to conduct ColourGrid surveys and analyses—this was undertaken by one co-investigator (CSX), who is an employee (CEO) of XAX Pty. Ltd. All research was conducted independently without influence by the funding bodies.

## Results

### Participation and demographics

Details regarding participation at each site are shown in (Table 2 and S3 Table, S3 and S4 Figs). Among 34,380 employees at the five sites, accurate HR data was available for 34,243 (99.6%; Exclusions: 36, no HCW category defined; 101, incorrect residential data). HR-derived HCW category information was consistent at all sites, except at Flinders Medical Centre where a small number of pharmacists and radiographers (n = 26; ≤ 0.001% of total dataset) were misclassified and could not be reconciled.

**Table 2:** Comparison of HR and PS data by participant demographics, study site, HCW categories and clinical-contact categories.

Features	HR Data (%)	PS Data (%)	*p-value*
	(n = 34 243)	(n = 1045)	
Mean age	42·3 years	43·4 years	0·0073
Female	25 909 (76%)	745 (78%)	NS
**Sites**			
Austin Hospital	7780 (23%)	321 (31%)	
Bendigo Hospital	3525 (10%)	165 (16%)	
Flinders Medical Centre	7395 (22%)	96 (9%)	
Monash Medical Centre	8303 (24%)	171 (16%)	
Westmead Hospital	7240 (21%)	292 (28%)	
**Healthcare worker category**			
SMO*	699 (2%)	103 (10%)	
VMO*	1635 (5%)	60 (6%)	
HMO*	2884 (8%)	90 (9%)	
Nursing	14878 (43%)	341 (33%)	
Ancillary	2797 (8%)	135 (13%)	
Administration / Clerical	4791 (14%)	157 (15%)	
Medical support	3636 (11%)	122 (12%)	
Hotel and Allied	2923 (8·5%)	37 (4%)	
**Clinical-contact category**			
Doctors	5218 (15%)	253 (24%)	<0·0001
Nurses—Allied Health	17675 (52%)	476 (46%)	<0·0001
Support Services	11350 (33%)	316 (30%)	0·049

*SMO—Full-time senior medical officer, VMO—Part-time senior medical officer, HMO—Hospital medical officer

ColourGrid surveys were completed by 1045 participants (Table 2; 3.05% total employees). The contribution of each site to both the HR data and the PS data are shown in Table 2 (also S3 Table and S3 Fig).

Overall, the demographics of survey participants were similar to employees in the HR database (Table 2), except that survey participants in the doctor and nursing-allied health categories were slightly older than those in the HR database (mean age: 42·4 *vs* 38·6 years, p<0·0001; and 43·2 *vs* 41·5 years, p = 0·0027, respectively; t-test). In the HR and PS databases, 75·6% and 77·6%, respectively, were women.

The distribution of HCW categories and CC categories were also broadly similar between the HR and PS databases (S3 Table and S4 Fig). Doctors made up a larger proportion of survey respondents (n = 253, 24%) relative to their contribution to the HR data (n = 5224, 15%). Overall, doctors were significantly more likely to participate in the survey than other CC categories—4·84% doctors *vs* 2·69% nursing-allied health *vs* 2·78% support services (p<0·0001; chi-square).

### HCWs compared to the Australian population

**Fig 2:**
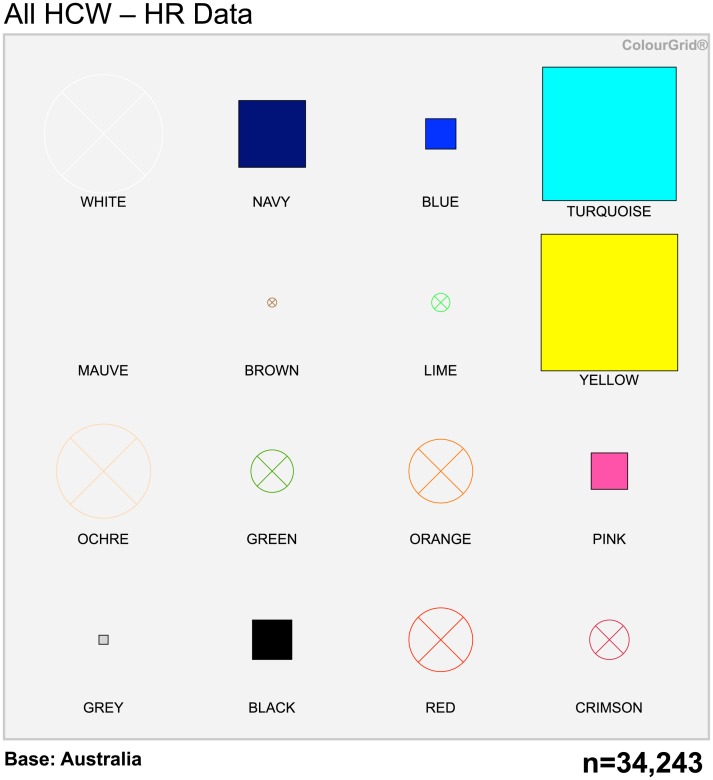
Projected personality profile of HCWs compared to the Australian population, based on HR data. HCWs were projected to have the following features: Higher than average levels of career minded professionals and post-secondary education; more affluent. Quick to take up new technology and new experiences. Very well informed, but often cynical about advertising messages and are generally difficult to convince. Assess issues then make their own decision. Challenging to others who do not share their interests or concerns to make a difference and leave a heritage of success.

The projected personality and marketing profile of HCWs based on HR data from all sites, compared to the Australian population, is shown in Fig 2 (with ColourGrid scores shown in S4 Table). These results were not predominantly due to Doctors/Nurses or any other specific HCW group, but were collectively influenced by all HCWs (data not shown). The results for each site separately are shown in S5 Fig Overall, compared to the Australian population, HCWs displayed more individualism, lower power distance and less uncertainty avoidance. They were considered likely to be quick to adopt new technology and new experience; more often cynical about advertising messages and were more likely to challenge others who did not share their interests or concerns to make a difference and leave a heritage of success.

### Comparison of results from HR and PS data analyses

**Fig 3:**
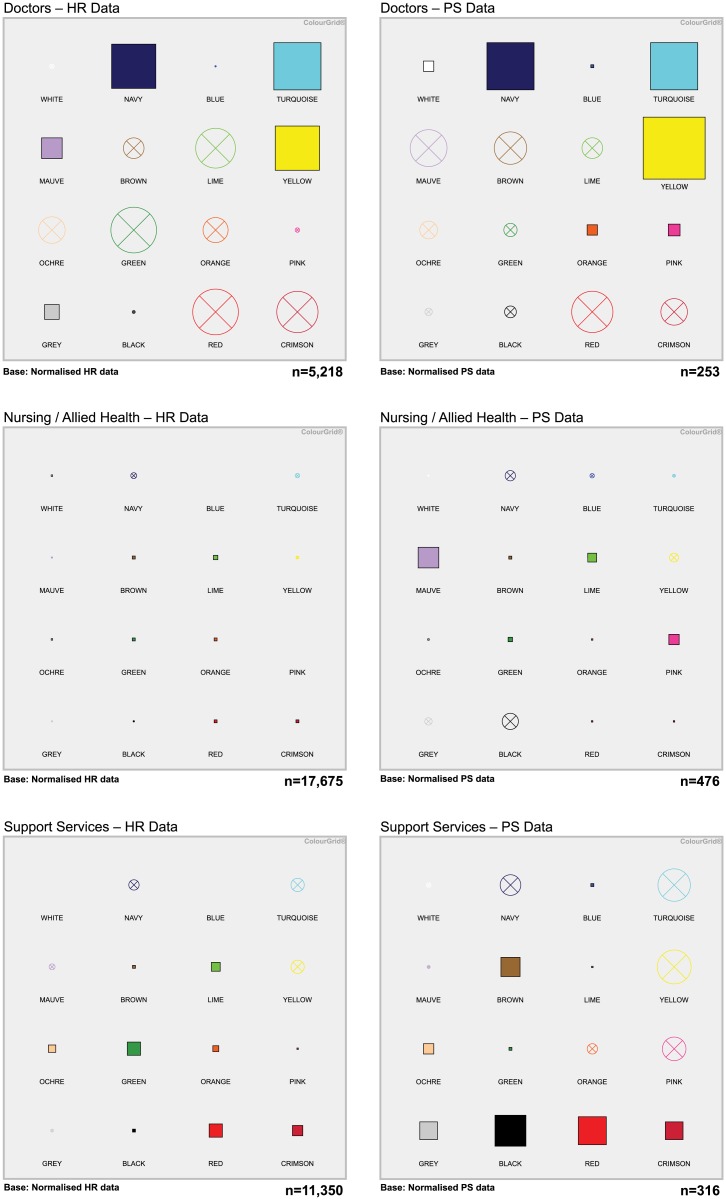
ColourGrid^®^ profiles for each HCW clinical-contact category based on HR and PS data. The number of HCWs analysed in each group (Doctors, Nursing-Allied Health, Support Services) are shown in the lower right-hand corner of each matrix. Derived personality profiles and messaging strategies are shown in Table 3.

ColourGrid profiles for the three CC categories derived from HR and PS data are shown in Fig 3, while the ColourGrid scores for all eight HCW categories and the CC categories are shown in S4 Table. Overall, ColourGrid profiles derived from HR and PS data were remarkably similar (Fig 3)–especially for doctors and nursing-allied health CC categories. For support services the PS data provided a similar overall colour matrix, but there was a slightly higher response in the grey-black region suggesting higher levels of uncertainty avoidance, insecurity and collectivism, than predicted by the HR data. The derived personality profiles and messaging strategies for each of the three CC categories are shown in Table 3.

**Table 3:** Derived personality profiles and messaging strategies for each HCW clinical-contact category based on the ColourGrid^®^ profiles shown in Fig 3.

HCW category	Personality profile	Interpretation and messaging strategy
**Doctors**	• Consider themselves independent and progressive thinkers—therefore feel that they should be able to act autonomously as they are well informed	• Independent thinkers—feel that they should be able to act autonomously as they are well informed
	• Don’t accept messages well and are generally cynical about hidden agendas	• Understand the intent of the rules—but are capable of rationalising why they do not necessarily need to follow them
	• Goal and vision-driven.	• Need direct personalised communication—cynical about blanket messages and hidden agendas
	• Need to highlight the individual positive and negative consequences to their adherence or non-adherence to the culture-change. Very alert to negative consequences of behaviour	• Need to highlight that adherence could make a positive difference and improve the future
	• Compliance governs behaviour	• “They are like cats—they are all independent and they believe they can do whatever they want and they believe they know what is best”
**Nursing-Allied Health**	• Balance their needs against the needs of others	• Engage them in the cause (the collective) as well as the behaviour (the individual)
	• Are focused on the present (not the past or future)	• Focus on the present.
	• Not exclusively information-driven—emotions and relationships play a big part in behaviour	• Interventions should focus on the immediate action and impact. “We can (need to) do it now”.
	• Have a comparable collective and individual response; the cause is collective, the behaviour is individual	• “Help us all get there together—we need to work as a team”
	• It’s about “them”, the team, rather than the individual	
	• Become highly committed once their emotion comes in—at that point it’s no longer about data	
	• It is important to engage them in the cause as well as the behaviour	
**Support Services**	• Not information-driven	• Are secure and comfortable when working within the rules
	• Are very comfortable with rules and like working within them.	• Protocolising the rules is important
	• The rules do not generally need justification	• Measuring against the rules is important
	• Rules provide certainty, especially when if one lacks knowledge	• Make the immediate manager responsible for each culture-change initiative
	• Don’t lack cognitive ability—just lack information	• Consequences for non-adherence work well—but failure to adhere should influence training, not be punitive
	• It’s what they’re not, rather than what they are. The mindset is therefore the antithesis of that of doctors	• Lack knowledge to make better choices, so if they are non-adherent then educate
	• Generally don’t want to make decisions and can’t make informed decisions (as they don’t have the knowledge)	
	• Highly collective and guided by their immediate manager—their immediate manager is the credible source of knowledge	
	• Work is generally not part of their life satisfaction. “I come to work so that I can live my life—my work is not my life”	

### Personality differences among doctor categories

**Fig 4:**
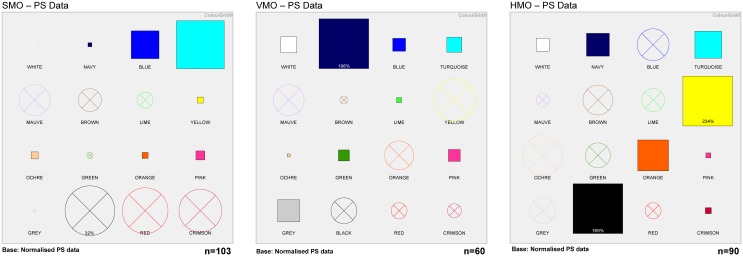
ColourGrid^®^ profiles for each of the three doctor categories based on PS data. The number of HCWs analysed in each group are shown in the lower right-hand corner of each matrix. Where box sizes >150%, the percentage is stated. Derived personality profiles and messaging strategies are shown in Table 4.

Analysis of PS data provided detailed profiles for each of the three categories of doctors—these are shown in Fig 4, with the derived personality traits and messaging strategies shown in Table 4. For VMOs and HMOs, where a number of features were >150%, the exact percentage is cited and the ColourGrid profiles with the relevant trait box drawn proportionate to the score is shown in S6 Fig Notable personality differences were identified between the three groups (Fig 4) which were likely to influence marketing strategies for culture-change initiatives (see below).

**Table 4:** Derived personality profiles and messaging strategies for each of the three categories of doctors based on the ColourGrid^®^ profiles shown in Fig 4.

Doctor category	Personality profile	Interpretation and messaging strategy
**SMOs**	• Can handle change and especially informed evidence-based change	• Highlight the evidence that guides the required change in behaviour
	• Feel that they are actively making an individual choice	• Establish a clear monitoring framework
	• Want to make good (correct) informed decision/choices based on evidence	• Need consistency between the evidence and the monitoring framework
	• Like measuring well defined outcomes or compliance measures—but these need to be managed and quantified	• If the framework is informative, adherence will be enhanced
		• Non-compliance is likely to be information-driven—therefore, punitive action for non-compliance is likely to be ineffective
**VMOs**	• “Affluential”–affluent and influential	• Set clear mandatory rules and mandatory monitoring
	• Personal reputation and prestige is highly important	• Need to understand that the requirement for compliance is inflexible (e.g. speed cameras)
	• Have a sense of authority and entitlement, supported by previous achievements	• Highlight that failure to comply may have negative consequences on their personal reputation
	• Concerned more about loss of prestige rather than gaining more	
	• Compliance with culture-change initiatives will not enhance their prestige as this has already been achieved	
	• Highly individualistic and exceptionalistic (“I am special”)	
	• Feel comfortable to not follow rules since “The rules are for everyone else”. “For me the rules don’t count as I have been doing this for so long”	
	• Good at ignoring rules unless non-compliance is associated with high consequence	
	• Having an evidence base for the rules is useful, but not sufficiently important to change behaviour	
**HMOs**	• Strong focus on future opportunities and career progression	• Compliance demonstrates leadership now and future potential
	• Compliance driven—need clear guidance about expected behaviour	• Highlight threat to future career by non-compliance
	• Knowledge and information drives their future	• Compliance provides an opportunity
	• Important positive concepts–“By following recommendations you will progress in your career”	
	• Affected by negative concepts–“Failure to comply, will lead to loss of career progression”	

SMOs—full-time senior medical officer; VMOs—‘visiting medical officers’, part-time senior medical officers; HMOs—hospital medical officers (see Table 1 for full descriptions)

### Application of personality profiling to specific infection control interventions

Based on the personality profiles derived from the study, suggested key intervention messages and marketing “tag lines” for each of the three infection control initiatives are outlined for the three CC categories and the three doctor categories in Table 5.

**Table 5:** Application of personality profiling to specific infection control strategies. Suggested key messages and marketing “tag lines” for each of the three infection control initiatives.

HCW Category	Key messages and suggested intervention “tag lines”		
	Hand Hygiene	Antimicrobial Stewardship	MRO Isolation
**Doctors—overall**	*“Hand hygiene appropriately—you know it’s right”*	*“Think about what’s needed—use antibiotics carefully”*	*“MRO isolation*?*—it’s too important—so follow the rules”*
		*“Re-assess the situation and prescribe appropriately”*	*“Isolation rules are important—so follow them and avoid the consequences”*
**Senior Medical Officers—Full-time (SMOs)**	*“The benefits of good hand hygiene in preventing hospital-acquired infections are indisputable”*	*“Antimicrobial prescribing should be rational with a clear indication*, *duration and expected outcome”*	*“Placing patients into isolation is inconvenient*, *but the risk of transmitting MROs to other patients is a much bigger issue”*
**Senior Medical Officers—Part-time (VMOs)**	*“Unless you do good hand hygiene*, *your reputation will suffer”*	*“Use of broad-spectrum antibiotics will have consequences and you will be held accountable for your actions”* ^1^	*“You will be monitored with mandatory reporting—so don’t risk your reputation”* ^2^
	*“Good hand hygiene is good medicine*. *Bad hand hygiene is bad medicine*. *No-one tolerates bad medicine*.*”*	*“You are smart*, *so prescribe appropriately”*	*“The bugs are smart too*, *so follow the isolation rules”*
	*“Good hand hygiene is good medicine*. *No-one tolerates bad medicine”*	*“Prescribe appropriately—or there could be problems”*	
**Hospital Medical Officers (HMOs)**	*“Realise your potential—perform good hand hygiene”*	*“Check and get antibiotic approval*, *you know your career is worth it”*	*“It’s easy to follow the isolation protocols and lead the way—don’t jeopardise your future“*
	*“Don’t wreck your future career by striking out on hand hygiene”*	*“Appropriate antibiotic prescribing shows your potential”*	
**Nurses-Allied Health**	*“Every time you hand hygiene*, *it shows you care”*	*“Antibiotic prescribing is a doctor’s responsibility*, *caring for the patient is yours”* ^3^	*“Don’t take the bugs in this room home with you—follow the rules”* ^4^
	*“Every 50 times you hand hygiene you save a life”*	*“Caring for your patients means it is OK to ask if the antibiotic is appropriate”*	*”Care for all your patients and follow the isolation rules”*
		*“Care for your patients—check if their antibiotics are appropriate”*	
**Support Services**	*“A good job needs good hand hygiene”* ^5^	Not applicable	*“Keep your job—follow the isolation rules”*
	*“Good hand hygiene is essential to doing a good job”*		*“Isolation rules*?*—just do it”*
	*“You know when to hand hygiene—so do it”*		

^1^ Antibiotic stewardship is likely to be difficult to enforce in this VMO mindset without individual prescriber monitoring, since identification of protocol breaches is critical to enforcement

^2^ Enforcement of isolation protocols in this VMO mindset will be difficult without clear objective evidence of non-compliance (e.g. video monitoring)

^3^ Nurses are disempowered regarding antibiotic prescribing as they don’t have the knowledge or power of the doctor. Thus, establishing a clear system of rules that gives nurses the authority to *not* act on behalf of doctors is likely to be effective.

^4^ Providing clear unambiguous rules and the reasons for the rules is important, but highlighting the emotional (and potentially dangerous) consequences of non-adherence will be very effective in this mindset; as will appealing to personal relationships.

^5^ Messaging requires clear unambiguous directives with no decision making required—*“Just tell me what to do and I will do it”; “It’s clear what you need to do—so do it”*

## Discussion

This study is notable given its large size (n>34,000) and its use of an innovative personality profiling tool that assessed HCWs based on their employment category—an approach that makes intuitive sense to any clinician working in a typical hospital. While previous studies have identified differences in behaviour, guideline adoption and beliefs between doctors, nurses and other HCWs, they have focused on small subsets of HCWs and not linked adherence to personality determinants [4,15,17,26–28]. Perhaps contrary to the assumptions of some policy makers and health bureaucrats, our findings suggest that Australian HCWs are rather different from the general population and that these differences (greater individualism, lower power distance, less uncertainty avoidance, cynicism about advertising messages) may be important for successful policy implementation. Furthermore, our data suggest that profiles based on non-identifying generic information that is obtainable from most hospital human resources departments is comparable in accuracy to that obtained directly from HCWs—especially for the three key CC categories of doctors, nurses and support services, where major personality differences were identified. Based on our data, it should be no surprise that many culture-change initiatives which generally employ a single approach to implementation, are associated with variable uptake by different HCWs, especially infection prevention interventions such as hand hygiene [5–8,26–28]. Such ‘market segmentation’ provides important primary research that may help guide development of more targeted interventions in a wide range of initiatives [12,14,25].

Similar to previous authors who have described a perceived lack of evidence or efficacy as one reason for doctors’ poor uptake of guidelines [1,3,11,26–28], our findings suggest that doctors require a personalised approach with a clear outline of the underlying evidence and the individual positive or negative consequences of their adherence to the intervention. Nursing-allied health staff, meanwhile, appear to be more concerned with outcomes as a collective group and are likely to connect with interventions that incorporate emotions and relationships rather than being purely information-driven. This is consistent with the findings from recent programs on guideline compliance in operating theatres and hand hygiene, where nurses emphasised the importance of the universal over the local, and focus on standardised approaches [9,12]. A focus on collective responsibility is also consistent with the recent Australian policy regarding control of antimicrobial prescribing and resistance in which all HCWs are expected to play a part [29]. Our data suggest that interventions for support services staff require a very structured framework of rules and regulations that are highly protocol-based and enforced by managers without requiring major intellectual justification. This group has traditionally been neglected in infection prevention literature, yet important interventions such as hospital cleaning are routinely performed by this group [4,30].

Importantly, we identified notable differences between doctors based on their seniority, and their full-time *vs* part-time hospital employment status—differences that are likely to be critical in explaining the shortcomings of some previous patient safety strategies [9,26,31] For instance, one Australian State recently introduced a “three strikes and you are out” hand hygiene policy whereby HCWs were threatened with periods of forced unpaid leave if they were observed to be non-compliant with hand hygiene on three occasions [32]. Our findings suggest that this approach would be ineffective (and considered offensive and disrespectful) for most HCW categories—thereby possibly explaining the policy’s failure to achieve medical or nursing endorsement [24]. Thus, insights from ColourGrid profiling may help predict whether interventions are likely to be successful or a waste of resources.

Although we applied our ColourGrid findings to guide three infection prevention strategies (Table 5), the same principles could be applied to many other HCW interventions—similar to other market segmentation approaches in the broader community [33].

This study has some limitations. Firstly, the study involved only HCWs resident in Australia, so we cannot be sure whether our findings will necessarily be relevant to HCWs in other countries, especially those with different healthcare structures. Nevertheless, it is likely that similarities exist across all nations in terms of the CC categories and probably also within categories of doctors. Secondly, our study involved only five Australian hospitals in three of the six states. However, given our national healthcare structure is standardised, especially in terms of HCW categories and their responsibilities, and our large study population, the findings are likely to be representative of the wider HCW population. Finally, we cannot be sure that our profiling conclusions and consequent recommendations regarding marketing strategies for each HCW group are totally accurate or indeed effective. For this reason, a subsequent study is planned to test these approaches in a blinded manner to quantify their efficacy. Nevertheless, our profiling results are a first step in providing a logical structure to culture-change implementation among a complex work force that many may have considered homogeneous.

We believe our study findings provide an innovative insight into personality differences among HCWs and how these characteristics, once recognised, can be exploited to develop intervention strategies that are more accurately targeted, while also potentially avoiding expenditure of resources on approaches that have little chance of success, or indeed, are likely to alienate certain HCWs. Development of an evidence-based framework or “blueprint” for HCW culture-change is likely to be more effective than the current approach.

## Supporting Information

S1 Fig Presentation of ColourGrid^®^ response scales. (DOCX)

S2 Fig ColourGrid^®^ survey questionnaire. (DOCX)

S3 Fig Comparison of HR and PS data submissions from each participating site (Fig A and B, respectively). (DOCX)

S4 Fig Comparison of Clinical-contact categories in the HR and PS datasets (Fig A and B, respectively). (DOCX)

S5 Fig ColourGrid^®^ profiles for each of the 5 study sites (comparator: Australia). (DOCX)

S6 Fig ColourGrid^®^ profiles for VMOs and HMOs derived from PS data where the relevant trait box is drawn proportionate to the score (see text for details). (DOCX)

S1 Table Typical features for each assigned colour trait in ColourGrid^®^. (DOCX)

S2 Table Three specific infection control initiatives with a summary of potential impacts of each on the HCW, the patient and the community. (DOCX)

S3 Table Total and site-specific HR-derived and PS-derived participation data. (DOCX)

S4 Table ColourGrid^®^ scores for HR-derived and PS-derived data. (DOCX)
