# Supplementary material for: Use of an Innovative Personality-Mindset Profiling Tool to Guide Culture-Change Strategies among Different Healthcare Worker Groups
Source: PLoS One. 2015 Oct 21;10(10):e0140509. doi: 10.1371/journal.pone.0140509 (PMC4619256; doi:10.1371/journal.pone.0140509)

**S-1 Fig. Presentation of ColourGrid^®^ response scales**

*Personality features more prominent than standard values:* These are represented as a solid coloured box in which the diagonal dimensions are linearly proportional to the identified value with a scale from 100% (standard) to a maximum of 150%. Where values are >150%, they are cited numerically below the coloured box.

*Personality features less prominent than standard values:* These are represented as a hollow coloured circles in which the radius are linearly proportional to the identified value with a scale from 100% (standard) to a minimum of 50%. Where values are <50%, they are cited numerically below the coloured circle.


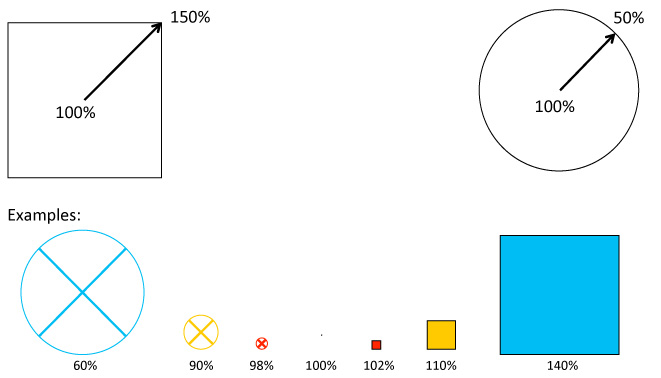

Supplement: S1 Fig — (DOCX) [file pone.0140509.s001.docx]
