# Supplementary figures and images for: Use of an Innovative Personality-Mindset Profiling Tool to Guide Culture-Change Strategies among Different Healthcare Worker Groups
Source: PLoS One. 2015 Oct 21;10(10):e0140509. doi: 10.1371/journal.pone.0140509 (PMC4619256; doi:10.1371/journal.pone.0140509)

**S-2 Fig. ColourGrid® survey questionnaire**


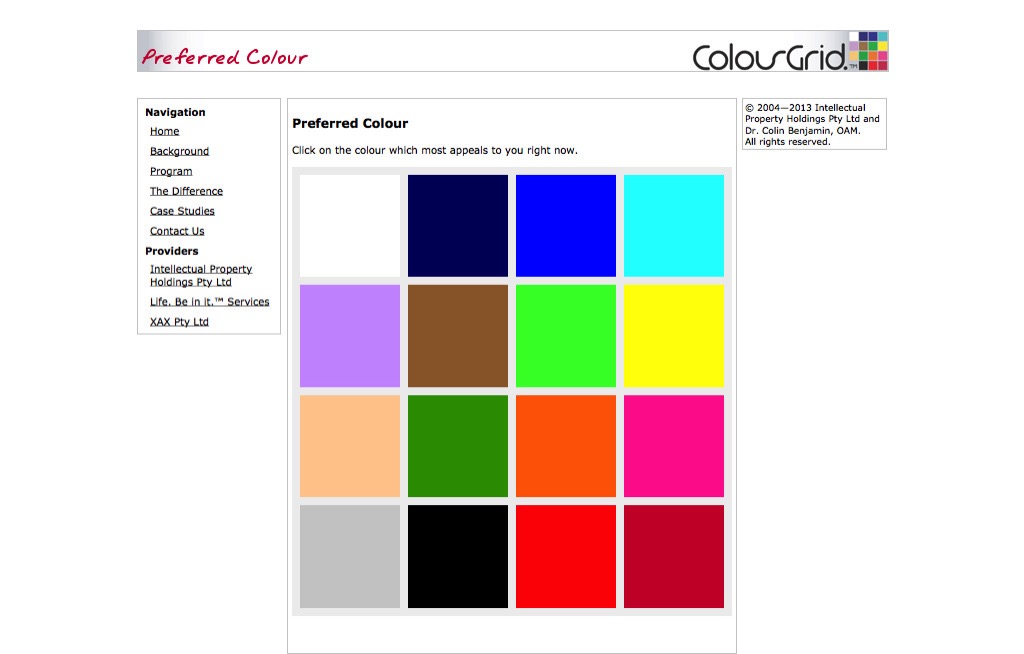


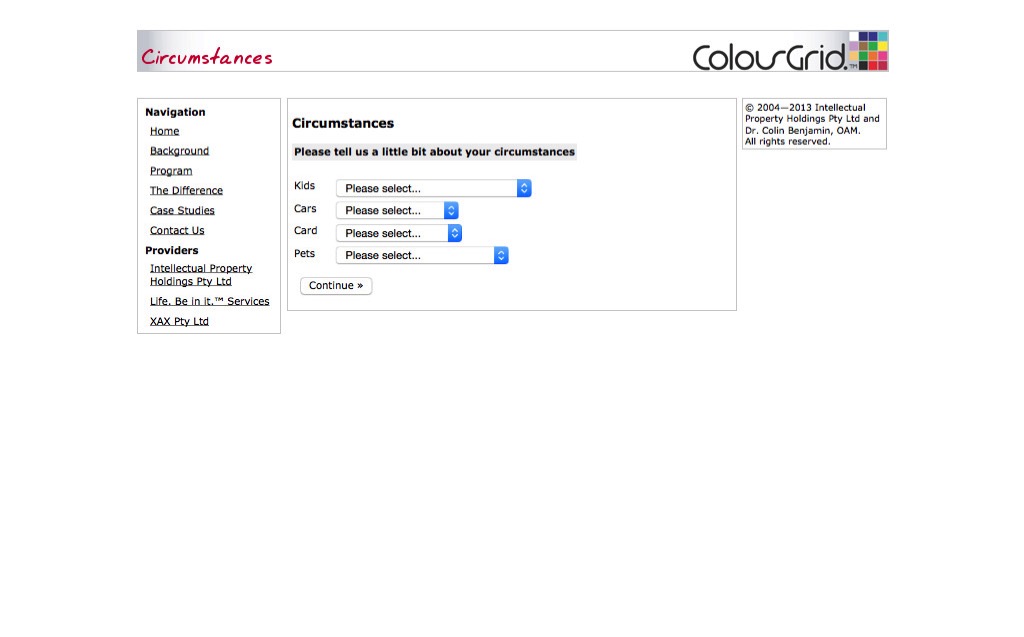


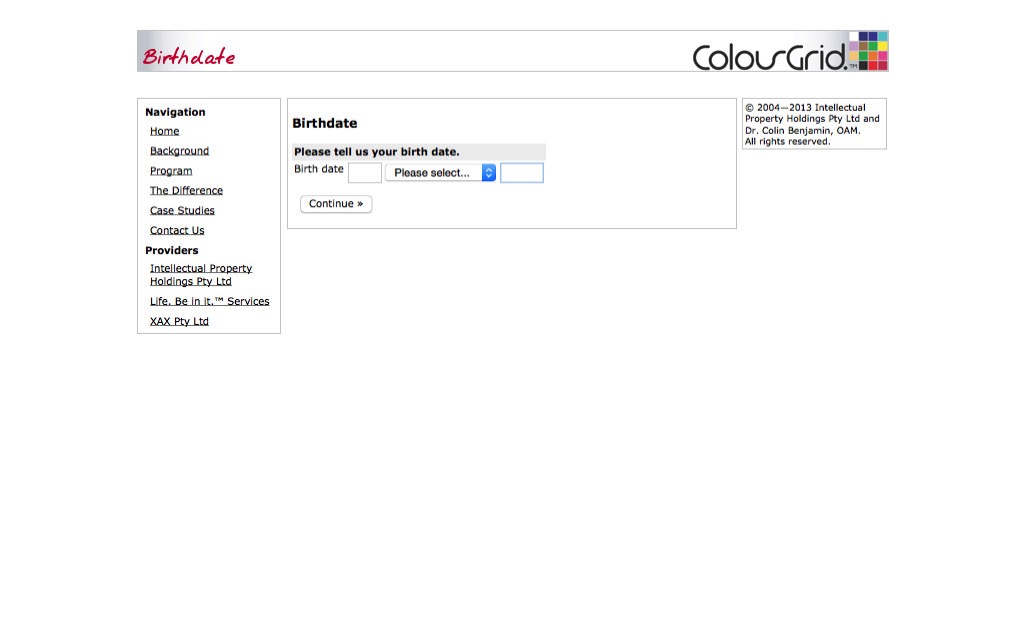


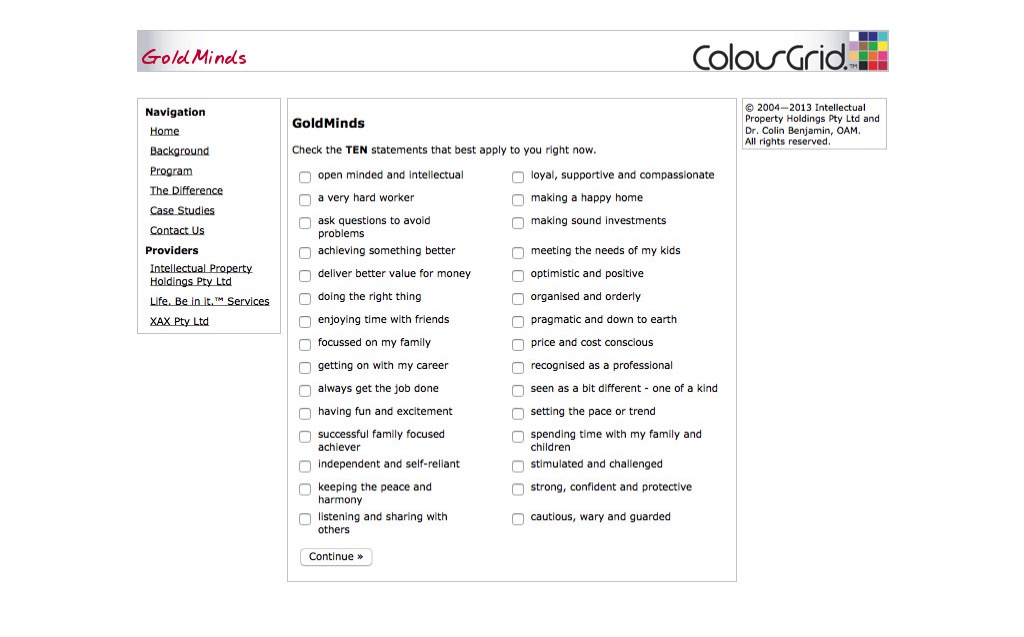


**
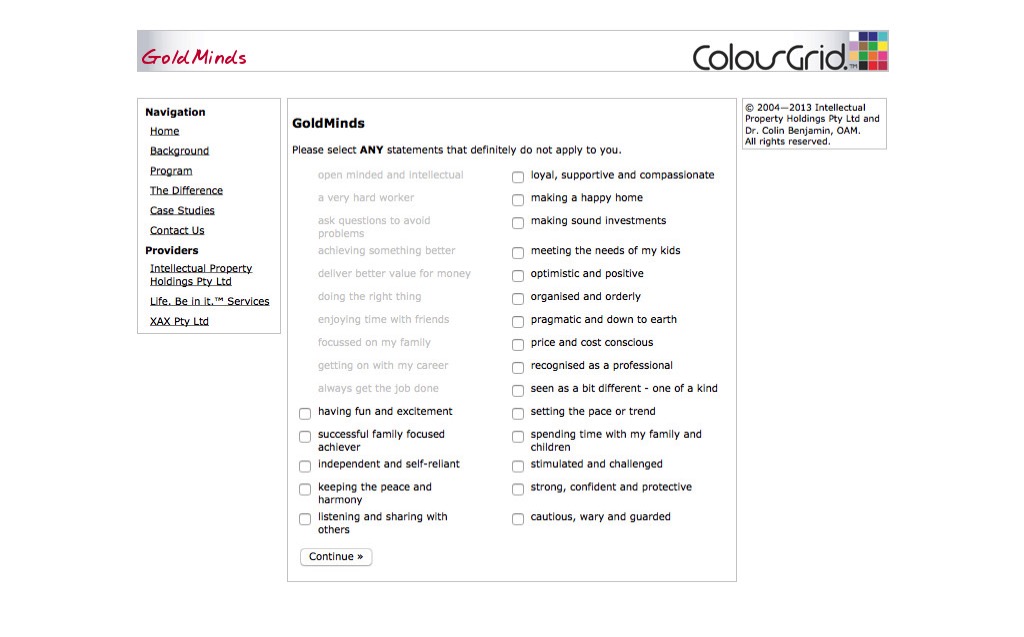
**

**
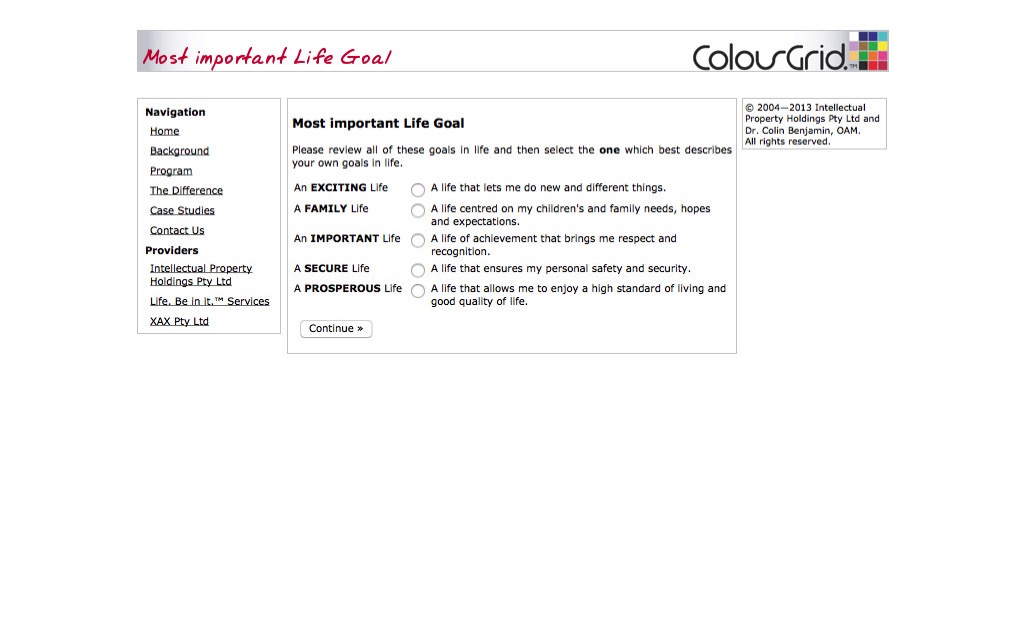

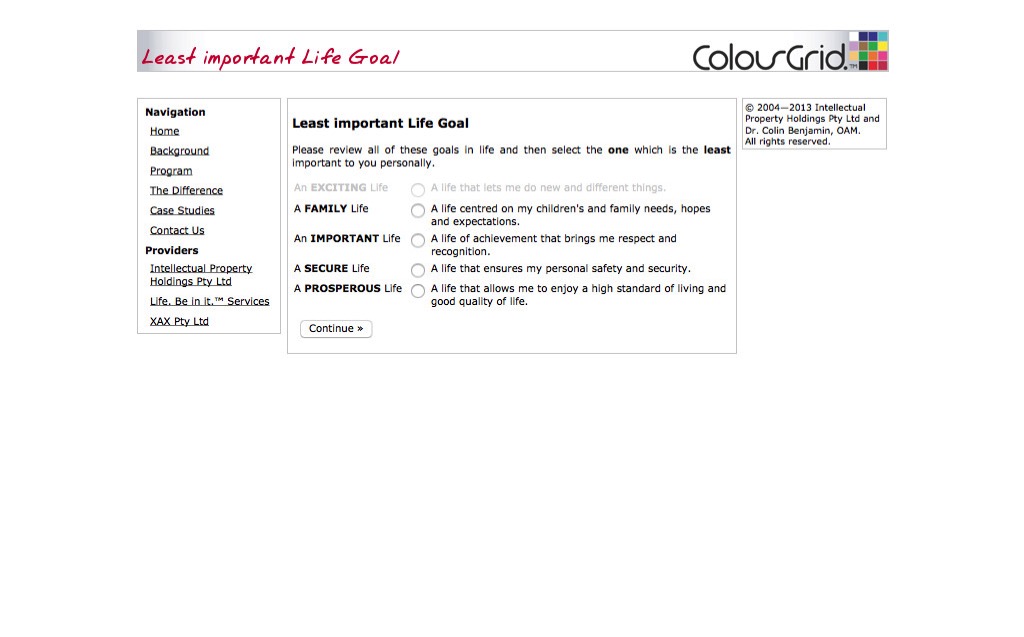
**

**
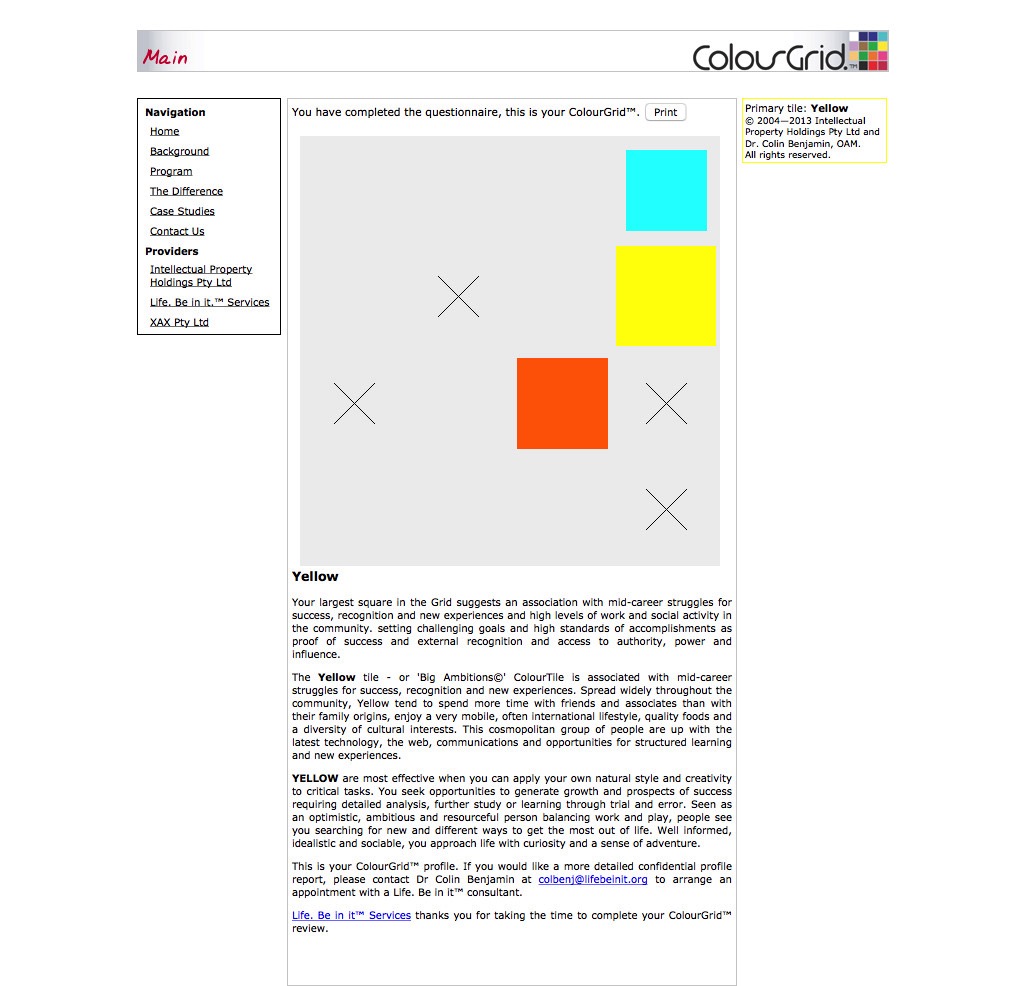
**

Supplement: S2 Fig — (DOCX) [file pone.0140509.s002.docx]
