# Supplementary material for: Use of an Innovative Personality-Mindset Profiling Tool to Guide Culture-Change Strategies among Different Healthcare Worker Groups
Source: PLoS One. 2015 Oct 21;10(10):e0140509. doi: 10.1371/journal.pone.0140509 (PMC4619256; doi:10.1371/journal.pone.0140509)
Supplement: S3 Fig — (DOCX) [file pone.0140509.s003.docx]

**S-3 Fig. Comparison of HR and PS data submissions from each participating site (Fig. A and B, respectively)**
