# Supplementary material for: Use of an Innovative Personality-Mindset Profiling Tool to Guide Culture-Change Strategies among Different Healthcare Worker Groups
Source: PLoS One. 2015 Oct 21;10(10):e0140509. doi: 10.1371/journal.pone.0140509 (PMC4619256; doi:10.1371/journal.pone.0140509)
Supplement: S4 Fig — (DOCX) [file pone.0140509.s004.docx]

**S-4 Fig. Comparison of Clinical-contact categories in the HR and PS datasets (Fig. A and B, respectively)**
