# Supplementary material for: Use of an Innovative Personality-Mindset Profiling Tool to Guide Culture-Change Strategies among Different Healthcare Worker Groups
Source: PLoS One. 2015 Oct 21;10(10):e0140509. doi: 10.1371/journal.pone.0140509 (PMC4619256; doi:10.1371/journal.pone.0140509)
Supplement: S6 Fig — (DOCX) [file pone.0140509.s006.docx]

**S-6 Fig. ColourGrid^®^ profiles for VMOs and HMOs derived from PS data where the relevant trait box is drawn proportionate to the score (see text for details).**
