# Supplementary material for: Use of an Innovative Personality-Mindset Profiling Tool to Guide Culture-Change Strategies among Different Healthcare Worker Groups
Source: PLoS One. 2015 Oct 21;10(10):e0140509. doi: 10.1371/journal.pone.0140509 (PMC4619256; doi:10.1371/journal.pone.0140509)
Supplement: S1 Table — (DOCX) [file pone.0140509.s007.docx]

**S-1 Table. Typical features for each assigned colour trait in ColourGrid^®^**

| **Colour** | **Title** | **Descriptors / Drivers** | **Long term orientation** | **Individualism / Collectivism** | **Power distance index** | **Uncertainty** | **Mindset** |
| --- | --- | --- | --- | --- | --- | --- | --- |
| **__** | Traditionalist | Cared for by others (eg. elderly, infirm, recovering from illness, overseas visitors) or responsible for the care of others. | Past | I | Them | No orientation | Taking care |
| __ | Private and privileged | Have made a very personal commitment to their community of interest, as a part of a well drilled, organised, well-off elite group of older wealthy individualists. | Past | I | Them | Acceptance | Cautious Investors |
|  | Premium successful lifestyle | Highly successful with more disposable income. Stronger desire for personal achievement and recognition. Heavy investment in the quality of both their work and leisure time performance. | Past | I | Us | Acceptance | Affluent Families |
|  | Seeking challenging and novel experiences | Higher than average levels of education. Very high quality of life. Highest socio-economic status and a very international or intellectually or orientated profile. | Present | I | Us | Acceptance | Trend developers |
|  | Comfort seekers | Conservative, highly structured lifestyle. Live away from other members of their immediate family. Transitioned from more active lifestyle to live a more satisfying and considered retirement. Enjoy security of regular and familiar choices. Need for integrity. Importance of standards or principles | Past | I | Them | Averse | Retired life |
|  | Cautious Investors | Cautious, considered and often very asset rich but income poor. Significant long-term investments. Risk avoidance, control. Strong religious / spiritual life. Focused on business developments. Need for integrity. Importance of standards or principles | Past | I | Them | No orientation | No risks |
|  | Value seekers | Family focused – children at home or primary school. Heavily committed to personal achievement and autonomy. Over-committed, time-stretched. Cautious and competitive. | Present | I | Us | Accept | Value hunters |
|  | Career progression | Developing career and struggling for success, recognition and new experiences. Seeking to gain acceptance for their ideas and contribution. Spend more time doing things with friends and colleagues than with their family origins. Mobile, often international lifestyle. Attracted to new technology. | Future | I | Us | Acceptance | Big ambitions |
|  | Conservative strivers; Empty nesters | Live vicariously through an extended family and community networks. One of largest and most rapidly growing segments as the number of children per household continues to decline. Suspicious of authority. | Past | We | Them | Averse | Empty nests |
|  | Co-operative and careful | Focus on a growing, healthy family. Struggling to balance needs and wants with tight budgets. Meeting needs of others. Innovative, creative and resourceful. | Present | We | Them | Averse | Happy homes |
|  | Fun seekers | Seeking to make a break from family demands. Wanting to develop capacity for independent living. Seeking excitement, instant gratification, new experiences and tastes. Reject pressure for responsibility and accountability. | Future | We | Us | No orientation | Fun lovers |
|  | Sharing the latest experience | Focus on education and mobility. Desire for new products. Instant gratification. Peer-driven but seeking to establish their own identity. | Future | We | Us | Acceptance | The “Tweens” |
|  | Survivors | Low levels of discretionary spending. Often single income households on government benefits or payments. Difficult choices about home, work and leisure opportunities. Careful with personal commitments. | Present | We | Them | Averse | Price conscious |
|  | Struggling | Family oriented, Coping with inadequate incomes, job insecurity and health concerns. Can be very defensive and hostile under pressure or feelings of getting an unfair deal in life. Desire control over their lives. Suspicious of authority. | Future | We | Them | Averse | Work is hard |
|  | Emotional, exciting | Families with school age children. New and unexpected pressures, especially around health and education concerns. Exciting family experiences. Autonomy. Strong emotional bonds. Pressed for time and disposable income. | Future | We | Us | No orientation | The youngsters |
|  | Gaining recognition | Families with young, pre-school children. New family demands. Meeting the needs of others. Heavily invested in early childhood development and better lifestyle chances. | Future | We | Us | N | Early years |
