# Supplementary material for: Use of an Innovative Personality-Mindset Profiling Tool to Guide Culture-Change Strategies among Different Healthcare Worker Groups
Source: PLoS One. 2015 Oct 21;10(10):e0140509. doi: 10.1371/journal.pone.0140509 (PMC4619256; doi:10.1371/journal.pone.0140509)
Supplement: S2 Table — (DOCX) [file pone.0140509.s008.docx]

**S-2 Table. Three specific infection control initiatives with a summary of potential impacts of each on the HCW, the patient and the community**

|  | **Hand Hygiene** | **Antibiotic stewardship** | **Isolation for MDROs** |
| --- | --- | --- | --- |
| **Health Care Worker** | - Simple | - Average complexity | - Complex |
|  | - High frequency | - Low frequency | - Low frequency |
|  | - Timing can be protocolized | - Timing critical | - Timing critical |
|  | - Low nuisance value | - High nuisance value | - Extreme nuisance value |
|  | - Personal adherence difficult to measure | - Personal adherence difficult to measure without electronic prescribing records | - Personal adherence difficult to measure without direct observation and feedback system |
|  |  | - Prescribing appropriateness difficult to measure unless matched to patient severity index |  |
| **Patient** | - Large individual impact | - Potential negative impacts in using narrow-spectrum agents in some conditions | - No benefit to individual MDRO-infected patient |
|  | - May be difficult to identify in specific patients | - Potential beneficial impact in terms of reduced adverse events (e.g. *C.* difficile) | - Risk of reduced care due to HCW nuisance value |
| **Community** | - Major cumulative benefits | - Cumulative benefits, but difficult to measure with many potential confounders | - Large immediate benefits |
|  | - Measurement generally based on multiple cross-sectional audits rather than individual reporting | - Difficult to match adherence with beneficial impact |  |
