# Supplementary material for: Use of an Innovative Personality-Mindset Profiling Tool to Guide Culture-Change Strategies among Different Healthcare Worker Groups
Source: PLoS One. 2015 Oct 21;10(10):e0140509. doi: 10.1371/journal.pone.0140509 (PMC4619256; doi:10.1371/journal.pone.0140509)
Supplement: S3 Table — (DOCX) [file pone.0140509.s009.docx]

**S-3 Table. Total and site-specific HR-derived and PS-derived participation data**

| **Site** | **Austin** | | **Bendigo** | | **Flinders** | | **Monash** | | **Westmead** | | **Total** | |
| --- | --- | --- | --- | --- | --- | --- | --- | --- | --- | --- | --- | --- |
|  | **HR data (%)** | **PS data (%)** | **HR data (%)** | **PS data (%)** | **HR data (%)** | **PS data (%)** | **HR data (%)** | **PS data (%)** | **HR data (%)** | **PS data (%)** | **HR data (%)** | **PS data (%)** |
| **HCW category** |  |  |  |  |  |  |  |  |  |  |  |  |
| SMO | 181 | 30 | 72 | 6 | 157 | 9 | 118 | 17 | 171 | 41 | 699 | 103 |
|  | (2%) | (9%) | (2%) | (4%) | (2%) | (9%) | (1%) | (10%) | (2%) | (14%) | (2%) | (10%) |
| VMO | 448 | 23 | 87 | 1 | 359 | 5 | 558 | 12 | 183 | 19 | 1635 | 60 |
|  | (6%) | (7%) | (2%) | (1%) | (5%) | (5%) | (7%) | (7%) | (3%) | (7%) | (5%) | (6%) |
| HMO | 701 | 30 | 218 | 10 | 682 | 4 | 654 | 16 | 629 | 30 | 2884 | 90 |
|  | (9%) | (9%) | (6%) | (6%) | (9%) | (4%) | (8%) | (9%) | (9%) | (10%) | (8%) | (9%) |
| Nursing | 3209 | 87 | 1626 | 71 | 3507 | 25 | 3484 | 79 | 3052 | 79 | 14878 | 341 |
|  | (41%) | (27%) | (46%) | (43%) | (47%) | (26%) | (42%) | (46%) | (42%) | (27%) | (43%) | (33%) |
| Ancillary | 739 | 38 | 317 | 27 | 698 | 14 | 430 | 17 | 613 | 39 | 2797 | 135 |
|  | (9%) | (12%) | (9%) | (16%) | (9%) | (15%) | (5%) | (10%) | (8%) | (13%) | (8%) | (13%) |
| Admin / Clerical | 1036 | 64 | 555 | 31 | 934 | 16 | 1427 | 18 | 839 | 28 | 4791 | 157 |
|  | (13%) | (20%) | (16%) | (19%) | (13%) | (17%) | (17%) | (11%) | (12%) | (10%) | (14%) | (15%) |
| Med support | 784 | 34 | 262 | 11 | 401 | 20 | 845 | 11 | 1344 | 46 | 3636 | 122 |
|  | (10%) | (11%) | (7%) | (7%) | (5%) | (21%) | (10%) | (6%) | (19%) | (16%) | (11%) | (12%) |
| Hotel & Allied | 682 | 15 | 388 | 8 | 657 | 3 | 787 | 1 | 409 | 10 | 2923 | 37 |
|  | (9%) | (5%) | (11%) | (5%) | (9%) | (3%) | (9%) | (1%) | (6%) | (3%) | (9%) | (4%) |
|  |  |  |  |  |  |  |  |  |  |  |  |  |
| **CC category** |  |  |  |  |  |  |  |  |  |  |  |  |
| Doctors | 1330 | 83 | 377 | 17 | 1198 | 18 | 1330 | 45 | 983 | 90 | 5218 | 253 |
|  | (17%) | (26%) | (11%) | (10%) | (16%) | (18%) | (16%) | (26%) | (13%) | (31%) | (15%) | (24%) |
| Nurses / Allied Health | 3948 | 125 | 1943 | 98 | 4205 | 39 | 3914 | 96 | 3665 | 118 | 17675 | 476 |
|  | (51%) | (39%) | (55%) | (60%) | (57%) | (41%) | (47%) | (56%) | (51%) | (40%) | (52%) | (46%) |
| Support services | 2502 | 113 | 1205 | 50 | 1992 | 39 | 3059 | 30 | 2592 | 84 | 11350 | 316 |
|  | (32%) | (35%) | (34%) | (30%) | (27%) | (41%) | (37%) | (18%) | (36%) | (29%) | (33%) | (30%) |
|  |  |  |  |  |  |  |  |  |  |  |  |  |
| **Total** | 7780 | 321 | 3525 | 165 | 7395 | 96 | 8303 | 171 | 7240 | 292 | 34243 | 1045 |
|  | (100%) | (100%) | (100%) | (100%) | (100%) | (100%) | (100%) | (100%) | (100%) | (100%) | (100%) | (100%) |

NB. Data are represented as No. (% of each site)
